# Intraoperative anaphylaxis due to aprotinin after local application of fibrin sealant diagnosed by skin tests and basophil activation tests: a case report

**DOI:** 10.1186/s40981-021-00472-6

**Published:** 2021-09-08

**Authors:** Masaki Orihara, Tomonori Takazawa, Tatsuo Horiuchi, Shinya Sakamoto, Mutsumi Uchiyama, Shigeru Saito

**Affiliations:** 1 Department of Anesthesiology, Gunma University Graduate School of Medicine, 3-39-22, Showa-machi, Maebashi, 371-8511 Japan; 2 Intensive Care Unit, Gunma University Hospital, 3-39-15, Showa-machi, Maebashi, 371-8511 Japan; 3 Department of Anesthesiology, Saitama Cancer Center, 780 Komuro, Ina-machi, Kitaadachi 362-0806 Japan

**Keywords:** Anaphylaxis, Aprotinin, Fibrin sealant, Skin test, Basophil activation test

## Abstract

**Background:**

There are few cases of anaphylaxis after local application of fibrin sealant diagnosed by skin tests.

**Case presentation:**

A 49-year-old woman underwent partial lung resection under general anesthesia. Anesthesia was induced uneventfully. Shortly after applying absorbable suture reinforcement felt that contained fibrin sealant, her systolic blood pressure fell to approximately 70 mmHg, along with facial flushing. Anaphylaxis was diagnosed based on the clinical symptoms and high serum tryptase levels. Three months after the event, skin tests were performed with all agents and were positive only for fibrin sealant vial no. 2, whose main component is aprotinin. Subsequently, basophil activation tests using fibrin sealant vial no. 2 and pure aprotinin demonstrated that the causative agent was likely aprotinin.

**Conclusions:**

We diagnosed aprotinin-induced anaphylaxis using skin tests and basophil activation tests. The occurrence of anaphylaxis should be considered when changes in vital signs are observed after the use of fibrin sealant.

## Background

Aprotinin is a serine protease inhibitor derived from the bovine lung that has been used widely as a component of biological sealants in surgical procedures [1, 2]. Aprotinin has been shown to reduce blood loss and transfusion requirements in various surgeries, including cardiac surgeries, organ transplant surgeries, and surgery for hip replacement [3]. Since its clinical introduction, aprotinin has been reported as a causative agent of perioperative anaphylaxis [1–4]. Although many cases of aprotinin-induced anaphylaxis have been reported, there are only a few cases of anaphylaxis due to aprotinin after the local application of fibrin sealant [2, 4]. In such cases, the diagnosis was mostly made by confirming aprotinin-specific immunoglobulin E (IgE) and/or immunoglobulin G (IgG) in the patient’s blood. To the best of our knowledge, this is the first report on a diagnosis of aprotinin-induced anaphylaxis using both skin tests and basophil activation tests (BATs).

## Case presentation

**Fig. 1:**
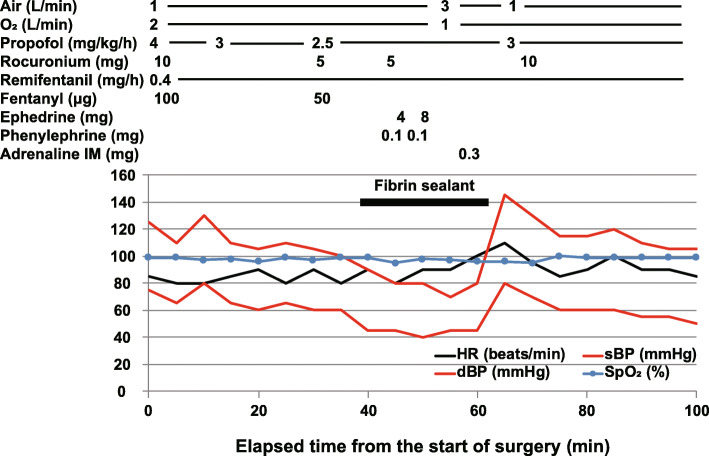
Drugs used during surgery and changes in vital signs. The horizontal black bar indicates the period of local application of fibrin sealant. IM, intramuscular injection; HR, heart rate; sBP, systolic blood pressure; dBP, diastolic blood pressure; SpO_2_, percutaneous oxygen saturation

Written informed consent for publication of this report was obtained from the patient. A 49-year-old, 59 kg, 159-cm tall woman with lung cancer underwent partial lung resection under combined general and epidural anesthesia. Approximately 1 year earlier, she had undergone the same surgery under combined general and epidural anesthesia and had been uneventfully exposed to aprotinin. After epidural catheterization using a standard procedure, anesthesia was induced with 70 mg propofol, 50 mg rocuronium, and 50 μg fentanyl. At the same time, continuous administration of propofol and remifentanil were started at the rate of 4 mg/kg/h and 1 mg/h, respectively. During the surgery, shortly after applying absorbable suture reinforcement felt made of polyglycolic acid that contained fibrin sealant, her systolic blood pressure fell to approximately 70 mmHg, along with facial flushing. Despite administration of a total of 12 mg ephedrine and 0.2 mg phenylephrine, her blood pressure did not recover (Fig. 1). Suspecting anaphylaxis, the felt was removed, and she was treated with intramuscular injection of 0.3 mg adrenaline and intravenous injection of 5 mg chlorpheniramine maleate and 50 mg ranitidine hydrochloride. Her general condition stabilized after these treatments (Fig. 1). The operation was completed successfully within 93 min. Since she did not show any respiratory symptoms at the end of anesthesia, she was transferred to the intensive care unit after extubation. The patient’s postoperative course was uneventful, and she was discharged home 18 days after the operation. Serum tryptase levels measured at the time of the reaction and 3 months after surgery were 13.4 μg/L and 1.1 μg/L, respectively. Based on the clinical symptoms and high serum tryptase levels, anaphylaxis was diagnosed. Her clinical score according to the consensus clinical scoring system developed by Hopkins et al. [5] was 7 points based on symptoms plus 12 points based on serum tryptase level, indicating a total of 19 points. Cases with a score of 15-21 points are judged as “very likely to be an immediate hypersensitivity reaction,” thereby supporting our diagnosis.

Three months after the event, skin tests with all agents, including the felt (Neoveil, Gunze Medical Japan Ltd., Osaka, Japan) and fibrin sealant (Bolheal, Teijin Pharma Ltd., Tokyo, Japan), were performed. The fibrin sealant kit (Bolheal, 3 ml) consists of four vials:

1. Lyophilized human fibrinogen (240 mg) and human factor XIII (225 units)
2. A solvent for fibrinogen and factor XIII containing the antifibrinolytic agent, bovine aprotinin (3000 KIE)
3. Lyophilized human thrombin (750 units)
4. A solvent for thrombin containing calcium chloride (17.7 mg).

We performed skin tests with all the vials except vial no. 4. Skin tests showed positive results only for fibrin sealant vial no. 2, whose main component is aprotinin (Table 1).

**Table 1:** Results of skin tests

Skin prick test						Intradermal test		
Drug	Concentration of the stock solution (mg/mL)	Judgment	Wheal (mm)	Flare (mm)	Concentration of the stock solution (mg/mL)	Judgment	Wheal (mm)	Flare (mm)
Saline	9	−			9	−		
Histamine	10	+	5	10	0.01	+	9	20
Propofol	10	−			1	−		
Rocuronium	10	−			0.1	−		
Remifentanil	0.05	−			0.005	−		
Fentanyl	0.05	−			0.005	−		
Cefazolin	20	−			2	−		
Flurbiprofen	10	−			1	−		
Lidocaine	10	−			1	−		
Ropivacaine	2	−			0.2	−		
Felt	NA	−			NA	ND		
Vial no. 1	80	−			8	−		
Vial no. 2	0.17	+	5	6	0.017	+	7	12
Vial no. 3	250^a^	−			25^a^	−		

^a^Units/mL

*SPT* skin prick test, *IDT* intradermal test, *NA* not applicable, *ND* no data

Saline and histamine were used as negative and positive controls, respectively. SPT was started with a 100-fold dilution of stock solution, and a tenfold dilution and stock solution were inspected. The SPTs for the felt were performed by puncturing a bifurcated needle from above the felt. Histamine and vial no. 2 showed a positive result at the stock solution. Subsequently, IDTs were performed. After confirming a negative reaction for saline and a positive reaction for histamine, vial no. 2 showed a positive reaction at a 100-fold dilution of the stock solution, that is, 0.00017 mg/ml. Other tested agents did not result in a positive reaction with a 100-fold dilution of the stock solution. SPT reactions were deemed positive when the diameter of the wheal was equal to or 3 mm larger than in the negative control, or equal to at least half that in the positive control after 20 min. IDT reactions were deemed positive when the diameter of the wheal was equal to at least twice that of the post-injection wheal after 20 min [6]

**Fig. 2:**
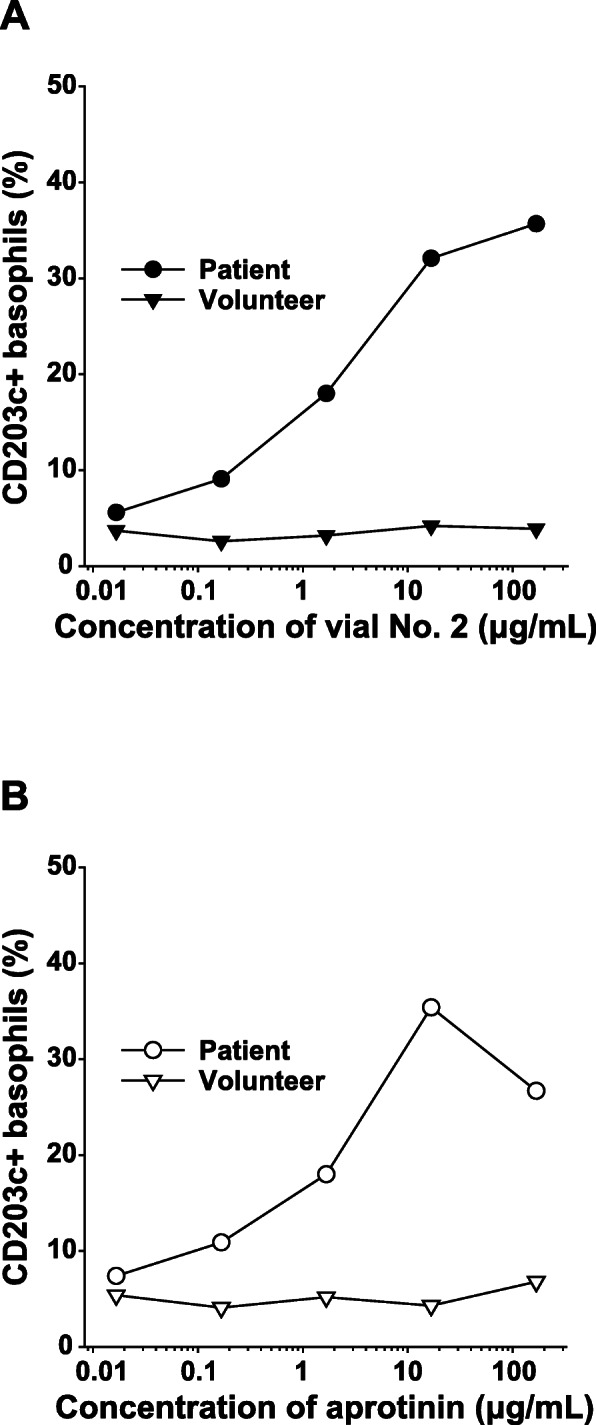
Results of basophil activation tests for CD203c. **A** Vial no. 2 induced CD203c upregulation in the patient but not in the volunteer. **B** Aprotinin induced CD203c upregulation in the patient but not in the volunteer

Subsequently, BATs for CD203c and CD63 were performed using serial dilutions of fibrin sealant vial no. 2 and purified aprotinin (Sigma-Aldrich Japan, Tokyo, Japan). The BAT was performed using a flow cytometer (FACSCanto II; BD Biosciences, San Jose, CA). An allergenicity kit (Beckman Coulter Inc., Brea, CA) was used for detecting CD203c+ and CD63+ basophils. Anti-IgE antibodies (10 μg/ml; Beckman Coulter) were used as a positive control. Compared to a healthy control, the patients CD203c+ basophils were significantly elevated to 35.7% and 35.4% by adding fibrin sealant vial no. 2 and purified aprotinin, respectively (Fig. 2). Similar results were obtained for CD63+ basophils (results not shown). These results demonstrated that the causative agent was likely aprotinin.

## Discussion and conclusions

We report here a case of anaphylaxis caused by aprotinin-containing fibrin sealant that was locally applied in the surgical field during pulmonary surgery. Skin tests and BATs showed that the causative agent of anaphylaxis was aprotinin.

Fibrin sealants have many different uses across a broad range of surgeries, where they have proved successful in controlling bleeding, providing suture support, and for tissue sealing. The common components of fibrin sealants are fibrinogen, thrombin, aprotinin, and calcium chloride [7]. Aprotinin has been identified as a causative agent of anaphylaxis, and many cases of aprotinin-induced anaphylaxis have been reported [2, 4]. Indeed, the incidence of hypersensitivity reactions was previously reported to be 2.8% on re-exposure to aprotinin, although the observed rate of hypersensitivity reactions on primary exposure is less than 0.1% [8]. However, there have been only a limited number of reports on anaphylaxis following the topical use of fibrin sealant [2, 4, 9–11]. This could be related to the fact that fibrin sealant is a substance used by surgeons in the surgical field and is difficult for anesthesiologists to recognize as a cause of anaphylaxis.

A recently developed clinical scoring system can be helpful to determine whether symptoms are due to anaphylaxis [5]. After the diagnosis of anaphylaxis, allergological assessment is essential to identify the causative agent and prevent recurrences. Skin tests remain the gold standard for detection of the culprit drug [6, 12, 13]. However, skin tests carry the risk of recurrence of anaphylaxis, and the positive predictive value of skin tests is not 100%. Hence, there seems to be room for other in vitro tests, including BATs, to diagnose anaphylaxis [14]. BATs recently became generally accepted as an additional and reliable tool with high sensitivity and specificity to identify the causative agent of perioperative anaphylaxis [14–16]. BATs have been used for testing for allergic reactions to a wide variety of drugs, including neuromuscular blocking agents, antibiotics, iodinated radiocontrast media, opiates, and sugammadex [17–20]. However, only few cases of aprotinin-induced anaphylaxis have been validated by BATs. Indeed, aprotinin-specific IgE and/or IgG were used for the diagnoses in the previous reports of aprotinin-induced anaphylaxis [2, 4]. In this case, we performed BATs in addition to skin tests to identify the causative agent, and similar results were obtained with both tests. These results suggest that both mast cells and basophils were likely involved in the underlying mechanism of anaphylaxis. BATs are probably helpful for the diagnosis of aprotinin-induced anaphylaxis. Further, the combination of BATs and skin tests allows for the diagnosis of anaphylaxis with high accuracy.

In conclusion, we diagnosed aprotinin-induced anaphylaxis using both skin tests and BATs. The occurrence of anaphylaxis should be considered when changes in vital signs are observed after the use of fibrin sealant in the surgical field during anesthesia.

## Data Availability

Data relevant to this case report are not available for public access because of patient privacy concerns, but are available from the corresponding author on reasonable request.

## Abbreviations

BATs: Basophil activation tests
IgE: Immunoglobulin E
IgG: Immunoglobulin G
